# Indigenious Races of the Earth; or, New Chapters of Ethnological Inquiry

**Published:** 1857-07

**Authors:** 


ART. III.—*Indigenous Races of the Earth; or, New Chapters of Ethnological Inquiry;* including Monographs on Special Departments of Philology, Iconography, Cranioscopy, Palæontology, Pathology, Archæology, Comparative Geography, and Natural History: contributed by ALFRED MAURY, Bibliothécaire de L'Institut de France; Secrétaire Général de la Société de Géographie de Paris; etc. etc. FRANCIS PULSZY, of Lubocz and Cselfalva, Fellow of the Hungarian Academy; Correspondent of the Instituto di Correspondenza Archeologica di Roma; Late Under Secretary of State in Hungary, etc., etc., etc., and J. AITKEN MEIGS, M. D., Professor of the Institutes of Medieine in the Philadelphia College of Medicine; Librarian of the Academy of Natural Sciences of Philadelphia; Recording Secretary of the Philadelphia County Medical Society; Fellow of the College of Physicians, etc. (With Communications from Prof. Jos. Leidy, M. D., and Prof. L. Agassiz, LL. D.) Presenting fresh Investigations, Documents, and Materials; by J. C. NOTT, M. D., Mobile, Alabama, and GEO. R. GLIDDON, Formerly U. S. Consul at Cairo, Authors of "Types of Mankind." Philadelphia: J. B. LIPPINCOTT & Co. London: TRUBNER & Co.

We have before us an advance copy of a book destined to make some stir in the scientific world, entitled, "Indigenous Races of the Earth." The authorship of this work is due to several hands. Dr. J. C. Nott, of Mobile, a distinguished ethnologist, contribntes much of the argument derived from external forms; Geo. R. Gliddon, formerly U. S. Consul at Cairo, adds some interesting chapters derived from the philology of nations; while Prof. Maury, the distinguished Hungarian philosopher Pulszky, J. Aitken Meigs, a professor in the Philadelphia College of Medicine, and Prof. Agassiz, also aid by the contribution of chapters of special subjects.

Several of these writers were engaged three years since in the production of that startling ethnological work, "Types of Mankind," and the present volume is intended to offer more comprehensive evidence of the doctrines then enunciated. The central idea of both volumes is, therefore, the same; and may be expressed in the proposition that the human race is not descended from a single pair, but that it, as well as all species of animal life, is the result of a series of creations, in nations or families, at different epochs. Thus the white races were created for the occupancy of the temperate zone, and associated with them were various families of animals, designed, also, with a view to their adaptation to special localities. So, too, the Negro, considered as the lowest form of humanity, is still specially adapted and created for the region in which we find him a native, and with him we find associated those higher forms of animal life, which most nearly resemble man. It is a remarkable fact in the distribution of animals, that the highest orders of monkeys are found inhabiting the same locality as the lower orders of Negroes.

The scope of this doctrine is evident. It brings the chronology of man in unison with that of the earth's surface, and assigns a period of 20,000 years before Christ as the shortest residence of the human race upon the globe. It recognizes a diverse origin for the different races of both man and animals; and this is not confined to the five great families, but is extended to many of their subdivisions which are supposed to have been created at different times: not in single pairs, but in nations.

If we accept this doctrine as scientific truth, many other contingent doctrines necessarily follow. We must believe that Adàm was not our common progenitor; that, therefore, accepting the Mosaic account of his fall, his sin could not have involved those races not lineally descended from him. Reaching further down the stream of time it lays a ruthless hand on other ideas dear to our sentiments, if not entirely consonant with our reason. The redemption through Christ, was devised to free mankind from the curse of Adam's fall, but if only a limited family of the human race were included in that curse, then the atonement fell short of the broad scope hitherto assigned it, and Christ died not for all, but for a small branch of the human family.

It is evident that a scientific theory like this, which reaches beyond and above the ordinary considerations of scientific truth, should not be accepted without close examination. Doubtless, all will be reconciled at last; the great fundamental truths on which not only our religion, but our civil and moral government are based, will stand immutable; but it were well to avoid the period of heresy and unbelief, of running after strange doctrines based on a half appreciation of the truth, which must intervene before the final agreement of intelligent opinion.

To this end it is necessary that all present discussion should be confined to the purely scientific question: Have the races of men and animals now resident on the earth a common origin at a certain point in Asia, from whence they were distributed throughout the world, or have they been created in their present form in the regions they now inhabit, and for which they are especially adapted?

The body of facts to which we must resort to solve this question, are to be found in what is at present known of the distribution of races and the effects of climate upon them. If we find that types of race are permanent, and that climatic changes do not effect any sensible or lasting change upon them, the position of Agassiz and his coadjutors will be sustained.

One great argument is to be derived from permanence of type. If we find that crossings and intermixtures of races, taken in connection with climatic influences, blot out the original type and substitute another for it, differing in essential particulars, then we may account for all the differences of race without supposing special origin for them. Opportunities for studying this are nowhere more abundant than in America, where we have a great variety of half-breeds.

Take, first, the most widely separated races—the white and the negro. Connections between them are prolific, but the offspring is in some sense a hybrid. The mulatto is a feeble existence, not fitted for any wide range of labor, or capable of much endurance. He is found when left to himself, not in the field, the workshop, or engaged in the inventive arts. He is employed in such duties as his physical nature permits, the menial duties of the household, and similar avocations. He is short-lived, presents a marked tendency to scrofulous and tuberculous disease, and the few children begotten by him develop still further this tendency. Few mulattoes reach anything like old age, and their families soon perish from the destructive elements generated by an improper cross. These peculiarities of the mulatto are not attributable to either origin, the white or the black, but simply to the fact that the races are physically so widely separated that an intermixture results in hybridity, as in the case of the horse and the donkey; and the deduction is, that whatever the original source, Adamic or diverse, the difference is now so wide as not to be overcome by amalgamation.

Taking now the white and American Indian races—more nearly allied than the white and negro—we find to a limited extent the same result. The Indian half-breeds are few in number, and do not perpetuate themselves to that full extent to be expected from the blending of two strong races. Indeed, so strong is that instinct of the white nature which regards these broad crosses of race as sinful, that amalgamation has never taken place to any great extent, except when the Indian has come in contact with the swarthy whites, the Gallic or Iberian Celt, in Central America, and the result of this union has been the production of a miserable and worthless race, incompetent to self-government, and with difficulty held above the level of savage life by the old Castilian element which still maintains its purity in those regions.

As we come to races nearly approximating each other, as the amalgamation of the Teutonic and Norman with the Celtic and Saxon elements, we have none of these evidences of hybridity. Prolificacy is not impaired, and the resulting offspring is hardy. Another wide difference is in the permanence of type. In unions of the white with the negro or Indian, we have a blending of the two without any tendency to revert to either of the original types; while in a mixture of the Saxon and Norman, for instance, we have a constant tendency to reproduce one or the other race. Of two children of such a union, the cross may be perceptible in neither, one child will be pure Norman, the other pure Saxon; and each will present, not only the physical, but the mental characteristics of its race. Where a mixture does take place in the first generation, and a blending of types results, it is only temporary; and in the second or third generation we shall see offspring presenting either the Norman or Saxon features in their purity.

The bearing of these facts, and others like them, is in favor of the new theory. Where the difference between races is wide, we have a hybrid offspring, the resulting cross is only temporary, and soon perishes from the elements of disease inseparable from it. Where the difference is narrow, and no hybridity follows, we have a constant reproduction of the type, a reversion to the original pattern. This is peculiarly marked in the Jewish race, which through all vicissitudes of climate and of government, and to some extent of amalgamation, still preserves the characteristic features which it had ages ago. The Grecian model of 3000 years ago, is still found unchanged in the Pelasgic nations, and though their daughters have for centuries filled the harems of the Turk and Arab, the latter are Turks and Arabs still.

In view of the short period assigned for the residence of man upon the earth, and of the fact that during three or four of the six thousand years no known changes of race have resulted from amalgamation, it is extremely difficult to assign the production of any new race to the blending of two preëxisting types. Indeed, could we do so, it would hardly help the argument; for we have then to account for the presence of two diverse races arising from the common Adamic stock. And again, if we are able to accomplish this, and account for the existence of the white and the black from climatic causes operating on the original Adamic stock, all that we have said heretofore, goes for nothing, and the Mosaic theory is maintained.

The question now comes up: What effect may climatic causes produce in modifying the complexion or features of a race?

The most obvious argument adduced is that the effect of a tropical sun is to darken the skin, and that it is possible that the perpetuation of this effect for ages might produce the blackness of the negro, while the gradual deterioration in mental capacity and physical conformation, consequent upon great heat, might also account for the thick lips, the curly wool, and the other peculiarities of the race.

Such a cause of diversity, to be truthful, should be constant. In the hot regions of Central Africa we find the negro, but we also find him in the temperate climate of the Cape of Good Hope; and again in the same latitudes of Asia we find a brown race, claiming great antiquity. On the continent of America we have one single race overspreading both temperate and tropical regions. The Esquimaux of the Polar regions is an exception, but he, too, is swarthy; and in the wilds of Canada we find the same complexion, straight hair, and other national characteristics, as on the line of the Equator.

Another argument brought by the advocates of the new theory is found in the distribution of animals. We have no space to go into an examination of the scheme of Agassiz, but it is evident that some startling facts are adduced. The anthropoid monkeys are found in some fifty varieties, differing very much in shape and intelligence. It is found that the same regions contain the highest forms of monkeys and the lowest forms of negroes, and that a gradual improvement is found in the human animal as we pass to lands where the monkeys deteriorate—that is differ more widely from the type of humanity.

Such are the arguments of this work on Indigenous Races. That they have force enough in themselves, and weight enough in the character of their advocates, to give them importance, cannot be denied. The present volume is an additamentum to "Types of Mankind," and consists of a vast medley of facts thrown together in the most admired confusion. The authorship is hydra-headed and argus-eyed, and each branch of the subject is a polyhedron. The result is that each eye of each head examines each side of the polyhedron, and a more inextricable tangle of logic and fact was never woven than this book presents. Aside from its real scientific merit, and the excellence of its typography, it is, in all its arrangements, the most villainous specimen of book-making on record.

For sale by Phinney & Co.

